# Kaempferol Improves Lung Ischemia-Reperfusion Injury *via* Antiinflammation and Antioxidative Stress Regulated by SIRT1/HMGB1/NF-κB Axis

**DOI:** 10.3389/fphar.2019.01635

**Published:** 2020-01-28

**Authors:** Chunli Yang, Wenkai Yang, Zhaohui He, Huiwei He, Xiaogang Yang, Yuanhua Lu, Hongbo Li

**Affiliations:** ^1^ Department of Intensive Care, Jiangxi Provincial People’s Hospital Affiliated to Nanchang University, Nanchang, China; ^2^ Department of Cardiovascular Surgery, Affiliated Central People’s Hospital of Zhanjiang of Guangdong Medical University, Zhanjiang, China

**Keywords:** kaempferol, lung ischemia-reperfusion injury, inflammation, oxidative stress, SIRT1, HMGB1, NF-κB

## Abstract

Trauma, organ transplantation, and thromboembolism are the main causes of lung ischemia-reperfusion injury (LIRI), and new therapies and drugs are urgent to relieve LIRI. In preliminary experiment, authors found that kaempferol could improve LIRI in rats, and the current study further explored its possible mechanism. The model of rat LIRI was established and appropriate research methods were implemented. Results shown that kaempferol could significantly improve LIRI, inhibit release of inflammatory factors including interleukin (IL) 6 and tumor necrosis factor (TNF) α in bronchoalveolar lavage fluid, and reduce oxidative stress reaction. Western blotting was used to detect protein expression levels and found that kaempferol could up-regulate the protein expressions of phosphorylated (p-) p65 and p65, and down-regulate the protein expression of sirtuin (SIRT) 1. Immunofluorescence was used to localize the expression of high mobility group box (HMGB) 1 and found its higher expression in outside of nucleus. However, the above effects of kaempferol on LIRI markedly attenuated by EX 527, a selective inhibitor of SIRT 1. Taken together, we first reported the protective effect of kaempferol on rat LIRI and confirmed that kaempferol’s antiinflammation and antioxidative stress involving the SIRT1/HMGB1/NF-κB axis.

## Introduction

Trauma, organ transplantation, and thromboembolism are the main causes of lung ischemia-reperfusion injury (LIRI). Ischemia triggers a series of hypoxic events leading to different degrees of cell damage and toxic substance release, and reperfusion can aggravate above effects, even more severe than ischemia (Laubach and Sharma, 2016). Ischemia-Reperfusion (I/R) can cause significant pulmonary inflammation, oxidative stress, and cell damage (Fernandez et al., 2013). This process will affect patient’s prognosis and even lead to death. Effective prevention and treatment are inadequate, and it is extremely important to find new therapies and drugs that relieve I/R injury (Bonservizi et al., 2014).

**Figure 1:**
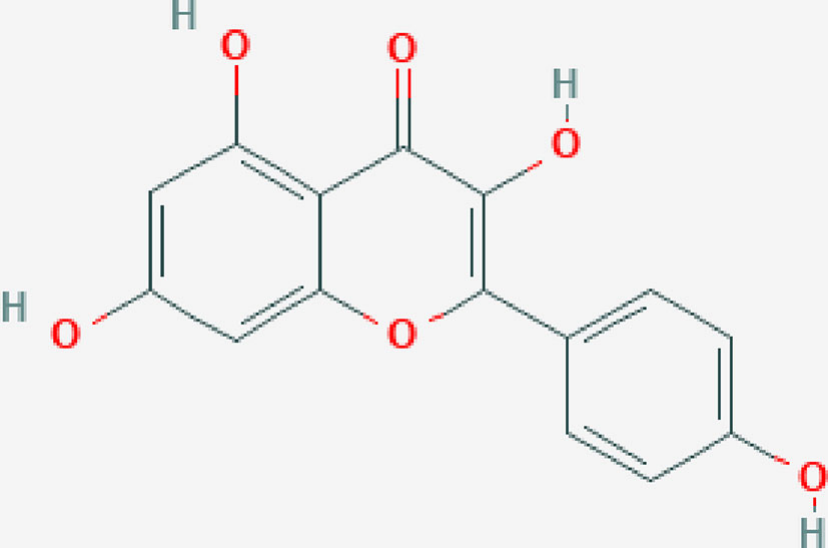
Chemical structure of kaempferol (Pubchem CID: 5280863).

Kaempferol (Kae, **Figure 1**) is a flavonol, which exists in many plants in nature, including a variety of fruits and vegetables that we eat in our daily life (Calderón-Montaño et al., 2011). Studies have shown that kaempferol has a variety of biological activities, including anticancer (Zhu and Xue, 2018), antioxidation (Saw et al., 2014), and antiinflammation (Devi et al., 2015). In the preliminary experiment, the authors found that kaempferol could improve lung injury induced by I/R in rats. Kaempferol is a potential natural drug to prevent or cure LIRI. Therefore, in present study, the authors further confirmed the protective effects of kaempferol on LIRI in rats, and explored its possible molecular mechanisms of action.

## Materials and Methods

### Reagents

Kaempferol (purity > 98%) was purchased from the National Institute for the Control of Pharmaceutical and Biological Products (Beijing, China), and dissolved in dimethylsulfoxide, with a final concentration in phosphate buffer solution (PBS). EX 527, also named Selisistat, was bought from the Sellectchem (Houston, TX, USA). Anti-sirtuin (SIRT) 1, anti-high mobility group box (HMGB) 1, anti-p65, and anti-phosphorylated (p-) p65 antibodies were acquired commercially from the Abcam (Cambridge, Cambs, UK), and anti-β-actin antibody were acquired from the Jiancheng Bioengineering Institute of Nanjing (Nanjing, JS, CHN). Myeloperoxidase (MPO), malondialdehyde (MDA), and superoxide dismutase (SOD) detection kits were purchased from Jiancheng Bioengineering Institute of Nanjing (Nanjing, JS, CHN). Interleukin (IL) 6 and tumor necrosis factor (TNF) α ELISA kits were purchased from Raybiotech (Peachtree Corners, GA, USA).

### Animals

Adult male Sprague Dawley (SD) rats, weighing 250–300 g, were obtained from the Nanchang University Laboratory Animal Center. The animals were allowed to access food and water ad libitum and maintained under a 12 h dark/light cycle at 22–25 °C. Experiments were carried out according to the Guide for the Care and Use of Laboratory Animals published by the U.S. National Institutes of Health (NIH Publication No. 85-23, revised in 1996), and approved by the Ethics Committee of Nanchang University (No. 2018-0125).

### Ischemia-Reperfusion (I/R) Protocol

Rats were anesthetized with pentobarbital sodium 50 mg/kg intraperitoneally and intramuscularly injected with 0.2 mg atropine. After disinfecting the skin of neck with iodophor, the subcutaneous tissue and muscle were dissected and separated, and the trachea was exposed with T-shaped incision. Trachea was intubated and mechanical ventilation was performed with 40% O_2_ at 45–55 strokes/min and a tidal volume of 8–10 ml/kg body weight. Open the chest at the left 3–5 intercostal space, free the left hilar, intravenous injection of heparin 50 U and arterial clip to block the left hilum for 90 min, then restore blood flow and reperfusion for 4 h. Anesthesia was maintained with inhaled halothane.

### Experimental Groups and Treatments

The rats were randomly divided into six groups with six rats in each as follows. (1) The sham group, the rats were performed thoracotomy, exposed the trachea and then closed the chest immediately. (2) I/R group, the rats were operated as above I/R protocol. (3) Low dose of kaempferol pre-treatment group [I/R + Kae (12.5 mg/kg)], the rats were intraperitoneally injected with 12.5 mg/kg kaempferol for 7 days before I/R operation. Surgery was performed as above I/R protocol immediately after the last administration. (4) Middle dose of kaempferol pre-treatment group [I/R + Kae (25 mg/kg)], operations were the same as I/R + Kae (12.5 mg/kg) group while the dose of kaempferol was 25 mg/kg. (5) High dose of kaempferol pre-treatment group [I/R + Kae (50 mg/kg)], operations were the same as I/R + Kae (12.5 mg/kg) group while the dose of kaempferol was 50 mg/kg. (6) SIRT 1 inhibitor intervention group (I/R + Kae +EX527), on the basis of I/R + Kae group, rats were intraperitoneally injected with EX 527 (5mg/kg, every 2 days, total 7 days before I/R operation), a SIRT1 selective inhibitor. The dose of EX 527 was confirmed in the pre-experiment. The rats in each group were sacrificed after re-perfusion by removing the ventilator. The lungs were removed from the thoracic cavity, and the right main bronchus was ligated. The left lung was perfused with 5 ml of pre-cooled sterile saline and recovered after 1 min. The recovery rate was not less than 70%. Repeat the above steps three times to obtain the total bronchoalveolar lavage fluid (BALF). Centrifugated at 3,200 xg for 10 min, harvested the supernatant and stored at -80 °C. One part of left lung after lavage was fixed with 4% paraformaldehyde. One part of left lung was weighed, dried at 60 °C for 48 h and weighed again to calculate the wet/dry (W/D) ratio. The remaining left lung was frozen directly at - 80 °C. **Supplementary Figure 1**.

### Assessment of Lung Injury

Paraffin section of lung tissue were prepared and stained by hematoxylin-eosin (HE) to evaluate lung injury through assessing the range of inflammatory infiltration and the thickness of the alveolar septa. MPO in the lung homogenate supernatants from the left lung was determined according manufacturer’s instruction (Jiancheng Bioengineering Institute of Nanjing, CHN), which was used to quantitate neutrophil accumulation in the lungs.

### Assessment of Oxidative Stress

The concentrations of MDA and SOD in left lung homogenate supernatants were detected according to manufacturer’s instruction (Jiancheng Bioengineering Institute of Nanjing, CHN).

### Assessment of Inflammatory Factors Level

Enzyme-linked immunosorbent assay (ELISA) was used to determine the production of IL 6 and TNF α in BALF according to the manufacturers’ protocols.

### Western Blotting

Western blotting was performed as previously (Yang et al., 2017). Briefly, a total of 30 mg protein from each tissue sample was separated and electrotransferred onto polyvinylidene difluoride membranes that was probed with primary antibodies (1:500) diluted in 5% nonfat milk and incubated at 4 °C overnight. After three washes with Tris-buffered saline and Tween 20 (TBS-T) for approximately 15 min, the membranes were incubated with the horseradish peroxidase-conjugated secondary antibodies. Then, the membranes were washed with TBS-T three times and each time lasting for 20 min. The immune complexes were visualized by enhanced chemiluminescence and the band intensity was measured quantitatively and analyzed with the Quantity One software (Bio-Rad, Pleasanton, CA, USA).

### Immunofluorescence

Briefly, after antigen retrieval, paraffin section (5 μm) were incubated with bovine serum albumin (BSA) followed by incubation with anti-HMGB 1 (1:200) for overnight at 4 °C. Then sections were washed by PBS for 3 times and each for 5 min. Sections were post-treated with Texas red-conjugated anti-mouse IgG (1:200; Abcam) for 1 h under the protection from light condition. Then washing sections by PBS for three times and each for 5 min. DAPI dyeing for 5 min, then washing and mounting. Inverted fluorescence microscope (Leica, Wetzlar, GER) was used to observe and photograph, and results were analyzed using Image Pro Plus 6.0 software (Media Cybernetics, Bethesda, MD, USA).

### Statistical Analysis

Data were presented as means ± standard error of the mean from six independent experiments and analyzed by SPSS version 20.0 (IBM Corp., Armonk, NY, USA). Two-tailed Student’s t-tests were performed to compare means between two groups. ANOVA followed by Bonferroni’s multiple comparisons was used to compare means from three or more groups. p < 0.05 was considered to be statistically significant.

## Results

### Effects of Different Doses of Kaempferol on LIRI

**Figure 2:**
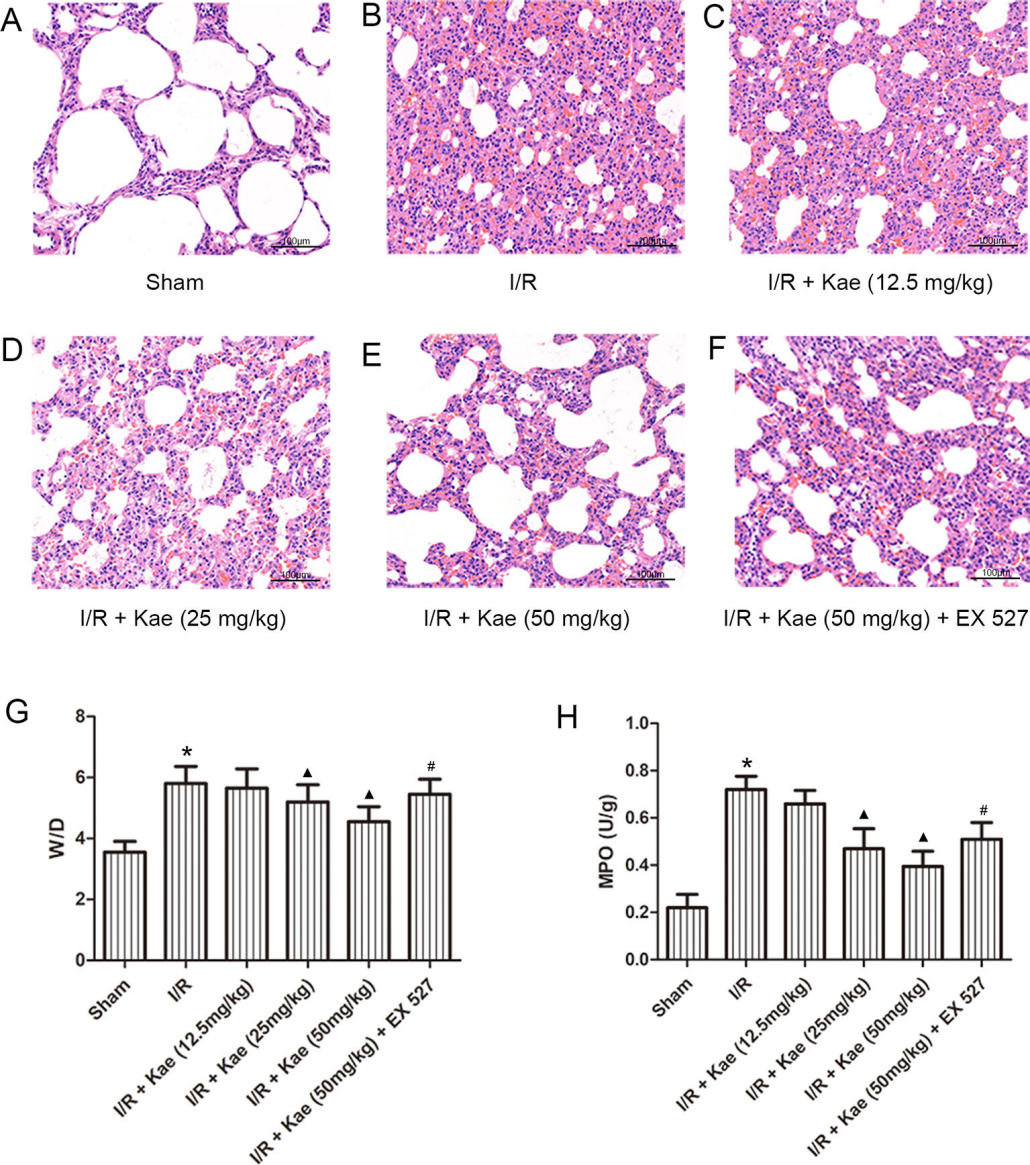
Effects of different doses of kaempferol on LIRI. **(A**–**F)** HE staining (200×). **(G)** The mean ratios of wet/dry in each group. **(H)** The mean concentration of MPO in each group. Data are presented as the means ± standard error of the mean for six independent experiments. *p < 0.05 vs. the sham group; ^▲^p < 0.05 vs. the I/R group; ^#^p < 0.05 vs. The I/R +Kae (50mg/kg) group.

HE staining (**Figures 2A–F**) results showed that after I/R, the lung tissue was severely damaged with alveolar structure was deformed, the epithelial cells were degenerated, necrotic and shedding, and interstitial edema was appeared. Inflammatory cells infiltrated, a large amount of fluid exudation and more red blood cells were seen in alveoli.

The W/D ratios of each group were shown as **Figure 2G**. Compared with sham group, W/D of I/R group was increased significantly (p < 0.05). With the increase dose of kaempferol, W/D decreased gradually, and the lowest W/D was found when kaempferol dose was 50 mg/kg.

The authors also measured the level of MPO in lung tissue. As shown in **Figure 2H**, compared with sham group, the concentration of MPO in lung of rats in I/R group increased significantly (p < 0.05). There was a negative correlation between different doses of kaempferol intervention and MPO concentration, among which 50 mg/kg kaempferol intervention had the lowest concentration of MPO.

Above results indicate that kaempferol could improve LIRI, and the effect was best when the dose of kaempferol was 50 mg/kg. Therefore, the subsequent experiments with kaempferol were performed at a dose of 50 mg/kg. What’s more, when compared with 50 mg/kg kaempferol intervention, the W/D and MPO were all significantly increased (p < 0.05) after addition with EX 527, a selective inhibitor of SIRT 1.

### Effect of Kaempferol on Oxidative Stress Reaction in Lung After I/R

**Figure 3:**
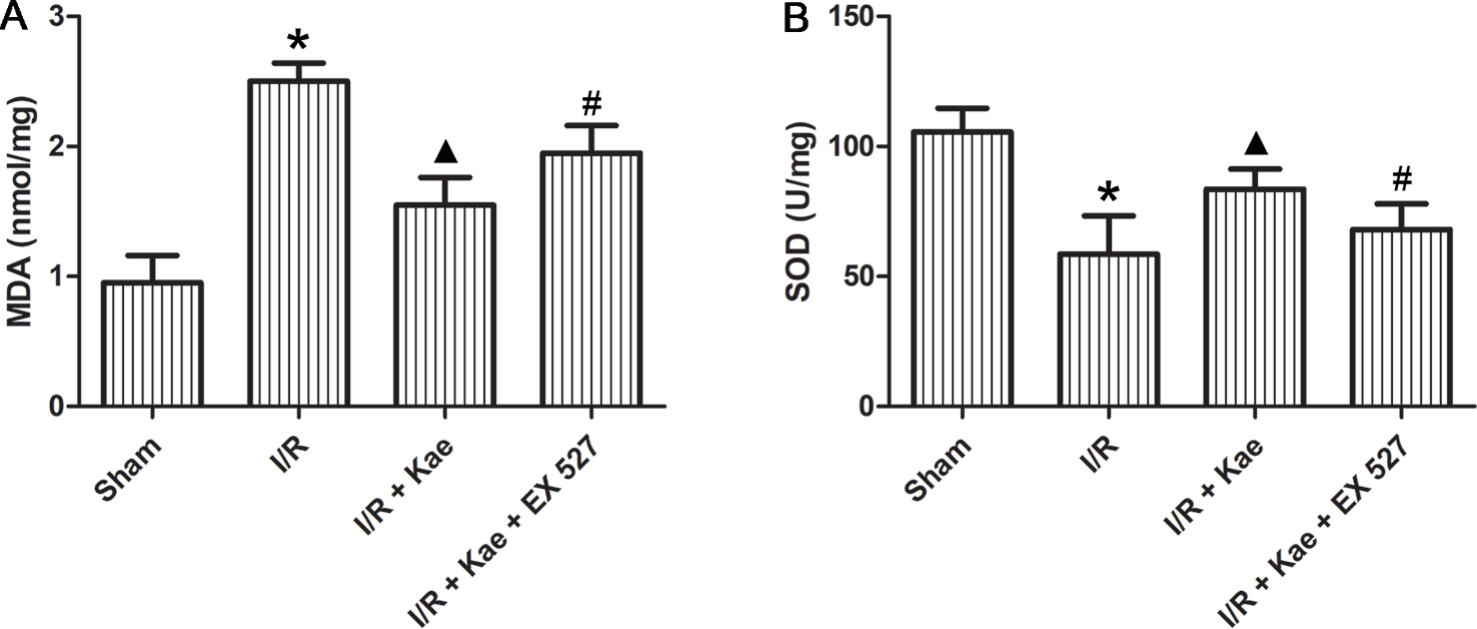
Effect of kaempferol on oxidative stress reaction in lung after I/R. **(A)** The concentration of MDA in lung of each group. **(B)** The concentration of SOD in lung of each group. Data are presented as the means ± standard error of the mean for six independent experiments. *p < 0.05 vs. the sham group; ^▲^p < 0.05 vs. the I/R group; ^#^p < 0.05 vs. The I/R +Kae group.

The levels of MDA and SOD in lung of rats in each group were detected. As shown in **Figure 3**, MDA level were significantly increased (p < 0.05) and SOD level were significantly decreased (p < 0.05) in the I/R group when compared to the sham group. Kaempferol could significantly decrease MDA level and increase SOD level (p < 0.05 vs. I/R group). However, above effects of kaempferol were significantly reversed by EX 527 (p < 0.05 vs. I/R + Kae group).

### Effects of Kaempferol on Release of Inflammatory Factors in Lung After I/R

**Figure 4:**
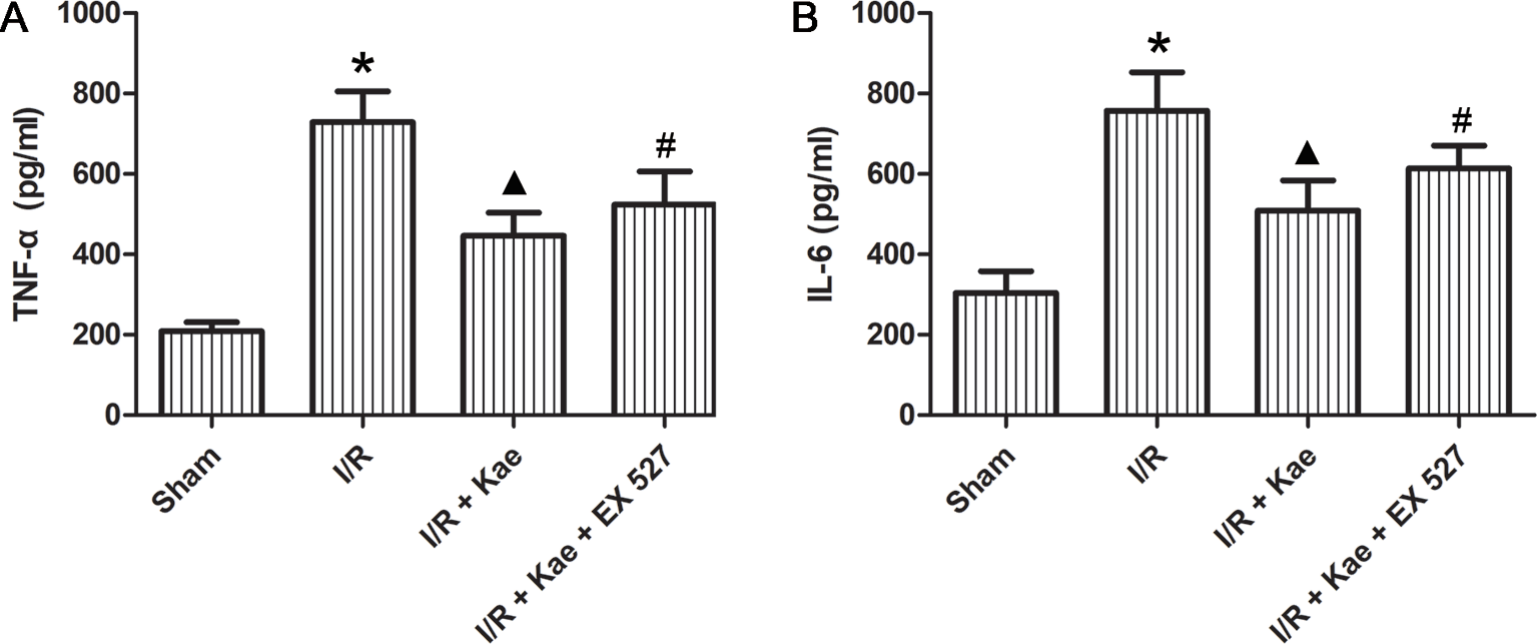
Effects of kaempferol on inflammatory factors in BALF. **(A)** The concentration of TNF α in lung of each group. **(B)** The concentration of IL6 in lung of each group. Data are presented as the means ± standard error of the mean for six independent experiments. *p < 0.05 vs. the sham group; ^▲^p < 0.05 vs. the I/R group; ^#^p < 0.05 vs. The I/R +Kae group.

ELISA was used to determine the levels of inflammatory factors in BALF. As shown in **Figure 4**, the release of IL 6 and TNF α in rat lungs increased significantly after I/R (p < 0.05 vs. sham group). In the group of I/R + Kae, the levels of IL 6 and TNF α were both decreased obviously when compared with I/R group (p < 0.05). However, the antiinflammation effect of kaempferol was significantly reversed by EX 527 (p < 0.05).

### Effects of Kaempferol on Expressions of P-P65 and P65 in Lung After I/R

**Figure 5:**
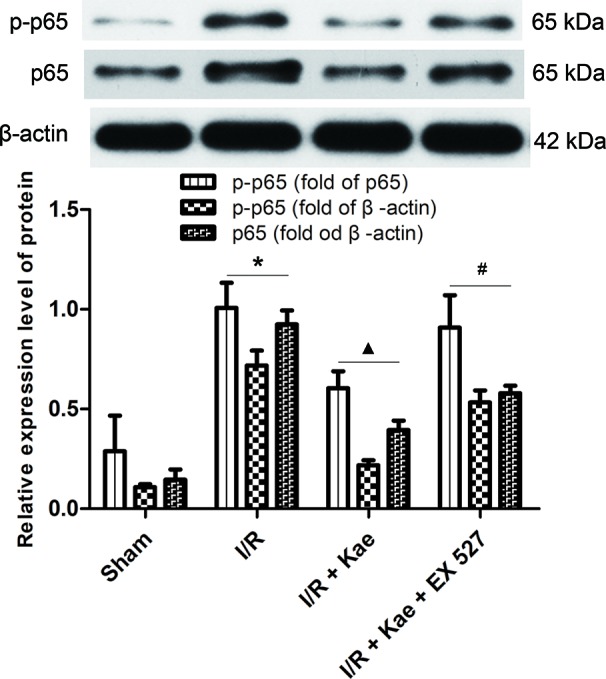
Effects of kaempferol on p-p65 and p65 expressions in lung after I/R. Data are presented as the means ± standard error of the mean for six independent experiments. *p < 0.05 vs. the sham group; ^▲^p < 0.05 vs. the I/R group; ^#^p < 0.05 vs. The I/R +Kae group.

As shown in **Figure 5**, the protein expression of p-p65 and p65 were both significantly increased in the group of I/R (p < 0.05 vs. Sham group). In the group of I/R + Kae, the expression level of p-p65 and p65 was obviously decreased when compared with the group of I/R (p < 0.05). However, the expression of p-p65 and p65 were both reversed in the group of I/R + Kae +EX 527 when compared with I/R + Kae group (p < 0.05).

### Effect of Kaempferol on Expression of HMGB 1 in Lung After I/R

**Figure 6:**
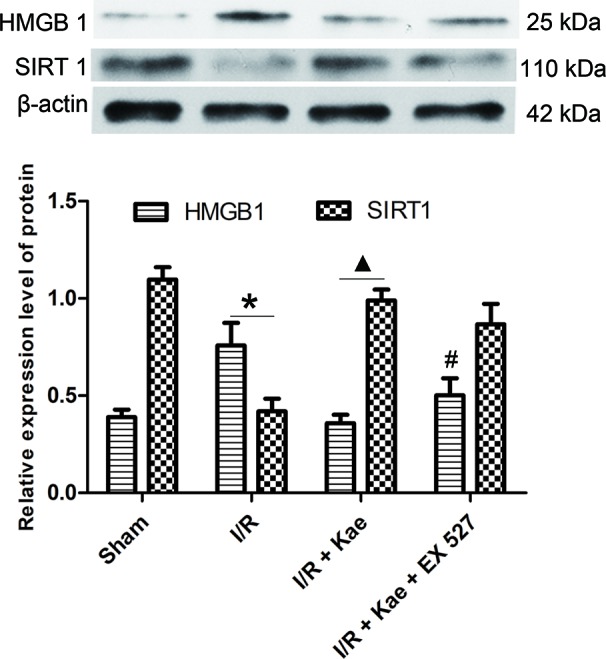
Effects of kaempferol on HMGB 1 and SIRT 1 expressions in lung after I/R. Data are presented as the means ± standard error of the mean for six independent experiments. *p < 0.05 vs. the sham group; ^▲^p < 0.05 vs. the I/R group; ^#^p < 0.05 vs. The I/R +Kae group.

The authors also detected the protein expression level of HMGB 1 in lung suffered I/R. The western blotting result shown in **Figure 6**. It shown that the protein expression of HMGB 1 was significantly up-regulated compared with the sham group (p < 0.05) while kaempferol could down-regulated protein expression of HMGB 1 significantly (p < 0.05 vs. I/R group). In the group of I/R + Kae + EX 527, the expression level of HMGB 1 was obviously up-regulated when compared with I/R + Kae group (p < 0.05).

**Figure 7:**
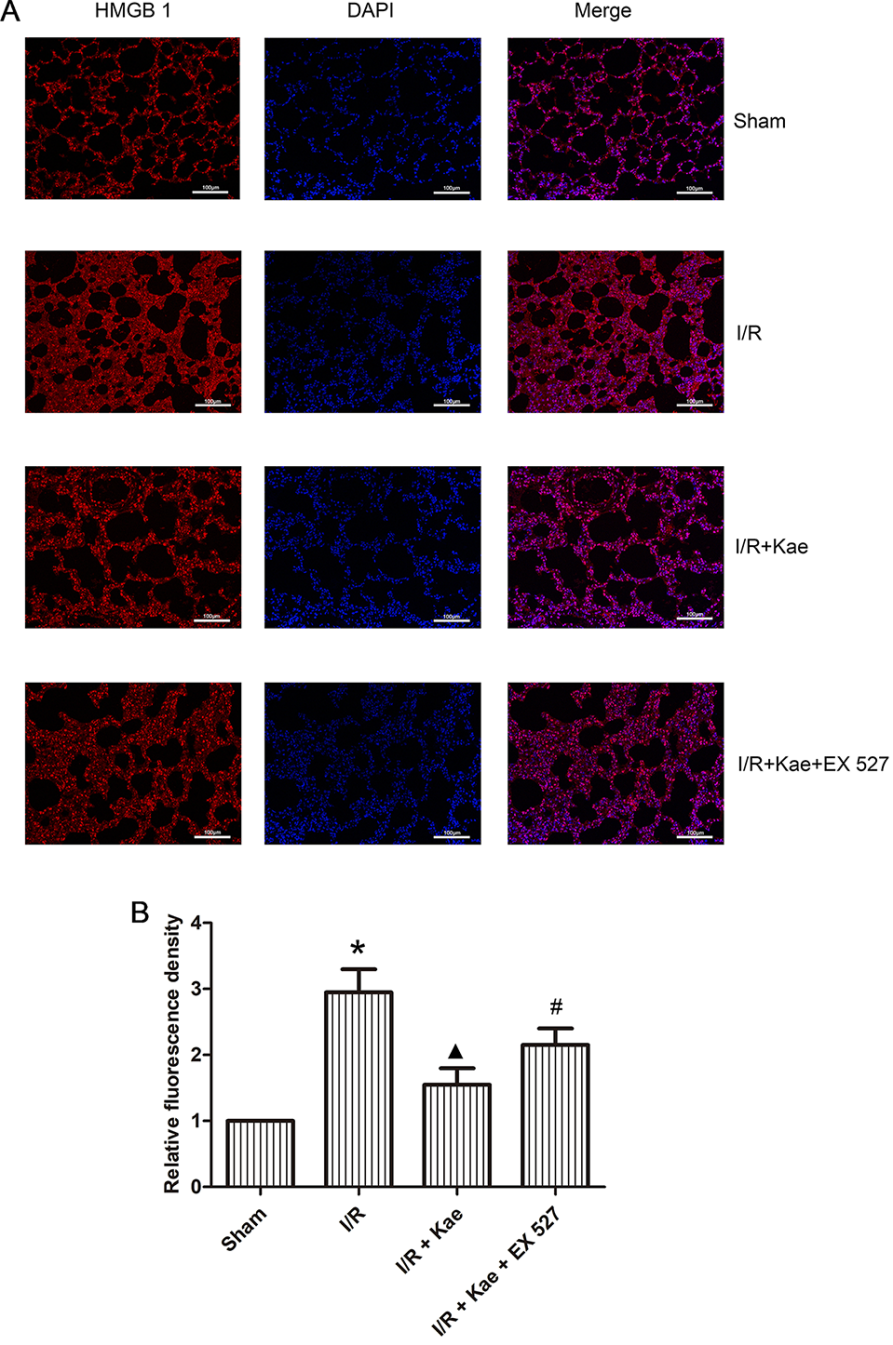
Effects of kaempferol on the distribution of HMGB 1 expression. Immunofluorescence staining was performed to observe the distribution of HMGB 1 expression. HMGB 1 protein was stained as red and nucleus was stained as blue. **(A)** Immunofluorescence staining of HMGB 1 in each group (200×). **(B)** Relative fluorescence density of HMGB 1 outside of nucleus in each group. Data are presented as the means ± standard error of the mean for six independent experiments. *p < 0.05 vs. the sham group; ^▲^p < 0.05 vs. the I/R group; ^#^p < 0.05 vs. The I/R +Kae group.

Immunofluorescence staining was performed to observe the distribution of HMGB 1 expression. As shown in **Figure 7**, the expression level of HMGB 1 outside of nucleus was increased obviously in the group of I/R when compared with the sham group (p < 0.05) while its expression level was reduced obviously in the group of I/R + Kae when compared with I/R group (p < 0.05). In addition, the expression level of HMGB 1 outside of nucleus was obviously increased when the activity of SIRT 1 was inhibited by EX527 (p < 0.05).

### Effect of Kaempferol on Expression of SIRT 1 in Lung After I/R

Western blotting was used to detect the protein expression level of SIRT 1 in lung of rats. As shown in **Figure 6**, the expression level of SIRT 1 was significantly down-regulated in the group of I/R when compared with the sham (p < 0.05). Kaempferol could significantly up-regulate SIRT 1 expression in lung suffered I/R (p < 0.05 vs. I/R group). However, the expression of SIRT1 in group of I/R + Kae +EX 527 did not change significantly, which may be related to the fact that EX527 could effectively inhibit the activity of SIRT1 deacetylase without interfering with its protein expression.

## Discussion

The present study shows that kaempferol could significantly improve I/R-induced lung pathological injury, decrease W/D ratio and MPO release, reduce oxidative stress, and inhibit the release of inflammatory factors. This is the first time to confirm the protective effects of kaempferol on LIRI. As a bioactive molecule, kaempferol has been widely studied in various lung-related diseases. Qian et al. found that kaempferol could inhibit LPS-induced acute lung injury in mice (Qian et al., 2019). Suchal et al. demonstrated that kaempferol has the effect of anti-myocardial I/R injury, and proved that it was associate with kaempferol’s ability to inhibit inflammation and apoptosis (Suchal et al., 2016). Du et al. reported that kaempferol can protect retinal pigment epithelial cells from oxidative stress injury through enhancing antioxidant activity (Du et al., 2018). Studies have shown that inflammation and oxidative stress are important causes of LIRI (Deng et al., 2015; Ferrari and Andrade, 2015), and the antiinflammation and anti-oxidative effects of kaempferol have been extensively reviewed (Devi et al., 2015; Onat et al., 2015). The authors of present study believe that the protective effects of kaempferol on LIRI was achieved through its antiinflammation and antioxidative effects.

Nuclear factor κB (NF-κB) is a major signaling pathway involved in the regulation of inflammatory mediators (Toby, 2009). When ischemia occurs, NF-κB translocates into the nucleus, then the pro-inflammatory factor genes are activated and followed by the release of a large number of inflammatory factors, including IL 6 and TNF α, which would further exacerbating organ injury (Wang et al., 2018; Qiu et al., 2019). In addition, NF-κB can also participate in the oxidative stress process *via* regulating the production of ROS and affecting the levels of SOD and MDA through nicotinamide adenine dinucleotide phosphate (NADPH) oxidase (Kleinschnitz et al., 2010). Many studies have shown that the antiinflammation and antioxidative effects of kaempferol are closely related to the regulation of NF-κB. Kaempferol can improve myocardial fibroblast inflammation through inhibiting NF-κB activity by regulating p65 and IκB α, the major related proteins of NF-κB signaling pathway (Tang et al., 2015). Kaempferol also can protect lung from acute injury in mice by inhibiting LPS-mediated NF-κB activation (Qian et al., 2019). In mouse retinal I/R injury, kaempferol suppressed NOD like receptor protein (NLRP) 1/NLRP3 inflammasomes and caspase-8 *via* c-Jun N-terminal kinase (JNK) and NF-κB pathways to attenuate retinal ganglion cell death (Lin et al., 2019). Kaempferol can reduce inflammation and oxidative stress reaction by inhibiting NF-κB nuclear translocation, and ultimately improve myocardial fibrosis and apoptosis caused by diabetes (Chen et al., 2018). In present study, the authors found that kaempferol could significantly reverse I/R-induced p-p65 and p65 up-regulation in lung, which suggested that kaempferol could inhibit p65 nuclear translocation. The protection effect of kaempferol on LIRI by regulating inflammation and oxidative stress may be through NF-κB signaling pathway.

HMGB1 is a highly conserved non-histone chromosome binding protein, which is closely related to a variety of lung diseases, including pneumonia (Tseng et al., 2014), tuberculosis (Zeng et al., 2015), chronic obstructive pulmonary disease (Sukkar et al., 2012), pulmonary fibrosis (Smit et al., 2014), and lung transplantation (Weber et al., 2014). Normally, HMGB1 is mainly concentrated in the nucleus. However, when cells are damaged or necrotic, lysine residues of HMGB1 are acetylated and migrated to the cytoplasm, and secreted to extracellular triggered by lysophosphatidylcholine as a result (Lu et al., 2013). Extracellular HMGB1 participates in the regulation of inflammation and oxidative stress through activating NF-κB by interacting with Toll receptor and receptors for advanced glycation end products (Bortolotto and Grilli, 2017). Present study showed that total and extranuclear HMGB1 were both significantly up-regulated after LIRI in rats, while their expression levels were significantly decreased after kaempferol administration, which suggesting that HMGB1 may be involved in the protection of kaempferol.

Studies have shown that SIRT1 can regulate the release of HMGB1 by deacetylation, thereby attenuating the inflammatory response (Hwang et al., 2015). SIRT1 is a NAD+-dependent class III protein deacetylase that participating in various metabolic and pathological processes *via* protecting cells against apoptosis, inflammation, and oxidative stress by regulating gene expression (Baur et al., 2012; Trovato Salinaro et al., 2018; Concetta Scuto et al., 2019). SIRT1 can directly participate in the regulation of inflammation through deacetylation and inhibit the transcription of inflammation-related genes (Zhang et al., 2010). Knocking down or knocking out SIRT 1 can increase the release of cytokines, while activating SIRT1 can significantly inhibit the expressions of TNF α, IL 8, and monocyte chemoattractant protein 1 (Yang et al., 2007; Dong et al., 2014). SIRT1-related pathways are also the core components of the redox signaling cascade. Alcendor et al. first reported the oxidative stress resistance of SIRT 1 *in vivo*. They found that overexpression of SIRT1 could protect mouse heart from oxidative stress induced by paraquat and increase the expression of antioxidants (catalase) (Alcendor et al., 2007). Since then, more studies have found that SIRT1 can regulate oxidative stress burden and its harmful effects (Hwang et al., 2010; Yuan et al., 2016). In present study, the authors found that the protein expression of SIRT1 was significantly attenuated in rat lung after I/R, while kaempferol could up-regulate its expression. To further confirm the regulatory role of SIRT1, EX527 was used to selectively inhibited the activity of SIRT1. As a result, the protective effects of kaempferol, including improvement of pathological injury, decrease of MDA and SOD levels, inhibition of inflammatory factor release, down-regulation of HMGB1, and the attenuation of NF-κB pathway activity, were all weakened. Based on the above results, the authors have evidences to believe that kaempferol may exert its anti-LIRI *via* SIRT1/HMGB/NF/κB axis.

In conclusion, the present study reported the protective effects of kaempferol on LIRI in rat including improving the pathological injury, inhibiting the release of inflammatory factors and reducing oxidative stress reactions. Further molecular biological studies have shown that the protective effects of kaempferol may be involve the SIRT1/HMGB/NF-κB axis. In addition, there are limitations to present study. Primary cells or cell lines was not applied to clarify detailed mechanisms of kaempferol. It is also not comprehensive to investigate the regulation role of SIRT1 signal only by inhibiting the activity of SIRT1. In further study, techniques of gene knockout or overexpression will be applied to further elucidate the role of the SIRT1 pathway in kaempferol anti-LIRI.

## Data Availability Statement

The scientific and statistical data used to support the findings of this study are included within the article. Requests for access to these data should be addressed to HL at hbli_nanchang@163.com.

## Ethics Statement****

The Present study was approved by the Ethics Committee of Nanchang University (No. 2018 - 0125).

## Author Contributions

CY and HL developed the original idea, designed the experiments, and edited and reviewed the final version of the article. WY, ZH, and HH performed experiments. XY and YL collated and analyzed the data. All listed authors contributed to article writing.

## Conflict of Interest

The authors declare that the research was conducted in the absence of any commercial or financial relationships that could be construed as a potential conflict of interest.

## Supplementary Material

The Supplementary Material for this article can be found online at: https://www.frontiersin.org/articles/10.3389/fphar.2019.01635/full#supplementary-material

Figure S1 Experimental design roadmap.
