# Supplementary figures and images for: Kaempferol Improves Lung Ischemia-Reperfusion Injury via Antiinflammation and Antioxidative Stress Regulated by SIRT1/HMGB1/NF-κB Axis
Source: Front Pharmacol. 2020 Jan 28;10:1635. doi: 10.3389/fphar.2019.01635 (PMC7025570; doi:10.3389/fphar.2019.01635)

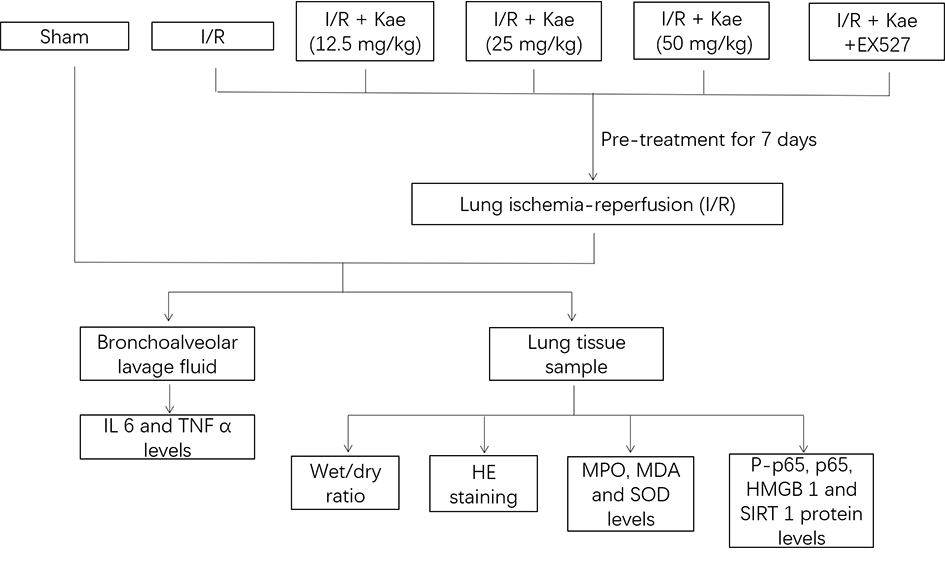

Supplement: Figure S1 — Experimental design roadmap. [file Image_1.tif]
